# Beyond metrics? Utilizing ‘soft intelligence’ for healthcare quality and safety

**DOI:** 10.1016/j.socscimed.2015.07.027

**Published:** 2015-10

**Authors:** Graham P. Martin, Lorna McKee, Mary Dixon-Woods

**Affiliations:** a University of Leicester, United Kingdom; b Aberdeen University, United Kingdom

**Keywords:** Patient safety, Healthcare quality metrics, Knowledge management, England

## Abstract

Formal metrics for monitoring the quality and safety of healthcare have a valuable role, but may not, by themselves, yield full insight into the range of fallibilities in organizations. ‘Soft intelligence’ is usefully understood as the processes and behaviours associated with seeking and interpreting soft data—of the kind that evade easy capture, straightforward classification and simple quantification—to produce forms of knowledge that can provide the basis for intervention. With the aim of examining current and potential practice in relation to soft intelligence, we conducted and analysed 107 in-depth qualitative interviews with senior leaders, including managers and clinicians, involved in healthcare quality and safety in the English National Health Service. We found that participants were in little doubt about the value of softer forms of data, especially for their role in revealing troubling issues that might be obscured by conventional metrics. Their struggles lay in how to access softer data and turn them into a useful form of knowing. Some of the dominant approaches they used risked replicating the limitations of hard, quantitative data. They relied on processes of aggregation and triangulation that prioritised reliability, or on instrumental use of soft data to animate the metrics. The unpredictable, untameable, spontaneous quality of soft data could be lost in efforts to systematize their collection and interpretation to render them more tractable. A more challenging but potentially rewarding approach involved processes and behaviours aimed at disrupting taken-for-granted assumptions about quality, safety, and organizational performance. This approach, which explicitly values the seeking out and the hearing of multiple voices, is consistent with conceptual frameworks of organizational sensemaking and dialogical understandings of knowledge. Using soft intelligence this way can be challenging and discomfiting, but may offer a critical defence against the complacency that can precede crisis.

## Highlights

• There are calls for greater use of ‘soft’ intelligence around quality and safety.
• Little research examines the challenges and opportunities soft data present.
• Our study in the English NHS found clinicians and managers saw utility in soft data.
• But dominant approaches to interpretation risked obscuring their greatest value.
• Soft data might better be used to disrupt understanding and challenge consensus.

## 1 Introduction

Egregious instances of healthcare system failure have occurred globally. They often reach public attention through an event involving a single patient—such as the death of Mary McClinton at the Virginia Mason Medical Centre in Seattle, USA (Kaplan and Patterson, 2008)—or sometimes through a catastrophe affecting many, such as the scandal of incompetent surgery at the Bundaberg Hospital in Australia (Van der Weyden, 2005). These kinds of healthcare crisis tend to share structural characteristics with other catastrophic events in demonstrating an unchecked drift into failure (Dekker, 2012). Disastrous incidents are characteristically preceded by an incubation period during which accumulating latent conditions and warning signs are ignored or misinterpreted due to habit and routine, where false assumptions and misplaced optimism predominate, and where attempted remedial action is misdirected (Macrae, 2014; Turner and Pidgeon, 1997). Partial or inaccurate information is often a crucial characteristic of the incubation period, but it is compounded by poor intelligence—the failure to seek out relevant data or interpret the available data properly. Cultures of denial, secrecy and protectionism, and fragmentation of knowledge about problems and responsibility for addressing them, are often implicated in such failures (Turner and Pidgeon, 1997).

Graphic, and tragic, illustrations of this pattern in healthcare include the recent cases in England of the Morecambe Bay maternity unit and Stafford Hospital. At Morecambe Bay, where poor care was implicated in the deaths of 11 babies and one mother, an independent panel identified a “repeated failure to examine adverse events properly” (Kirkup, 2015, p.183). “High-level metrics,” the report added, “may not be sensitive to the underlying risks. For that reason, it is important to understand what is happening in clinical services themselves” (Kirkup, 2015, p.51). At Stafford, a public inquiry showed that systems of regulation and oversight failed to identify and remedy problems of quality and safety over a long period, linked to a preference among hospital leaders for information that appeared to reflect well upon the organization and a corresponding tendency to discount discomfiting data. “Statistics and reports were preferred to patient experience data,” the inquiry found (Mid Staffordshire NHS Foundation Trust Public Inquiry, 2013, p.13). To prevent future similar tragedies, the inquiry recommended that hospitals take greater heed of the insights of staff, patients and carers, through “greater attention [ … ] to the narrative contained in, for instance, complaints data, as well as to the numbers” (Mid Staffordshire NHS Foundation Trust Public Inquiry, 2013, p.90).

Subsequent efforts to identify and address problems of quality and safety of care in England have counselled an emphasis on ‘problem-sensing’ rather than ‘comfort-seeking’ approaches (Dixon-Woods et al., 2014). Problem-sensing should be geared towards detecting fallibilities in organizational systems and cultures that might lead to disasters, creating the “chronic unease” (Fruhen et al., 2015, p.969) thought to be important in assuring safety other high-risk industries. Hard metrics (e.g. rates of infections and complications) will continue to play an invaluable role in identifying hazards and risks in healthcare organizations, perhaps coupled with proactive risk detection tools. Yet as experiences at Stafford, Morecambe Bay and elsewhere demonstrate, hard data, valuable as they are, may not yield full insight into the range of vulnerabilities and fallibilities in organizations.

One reason for this lies in the social practices that influence the production of such data. What gets recorded and reported is not the outcome of a neutral scientific process; rather, it reflects both the raw material available to those doing the recording and the decisions of official authorities about the classification of that material (cf. Bloor, 1991). Acts of recording and determination themselves take place in organizational contexts where actors are attentive to the possible uses to which data may be put (Kitsuse and Cicourel, 1963; Dixon-Woods et al., 2012), such that official records may provide a distorted picture or diminish important particularities of context. Official sources of knowledge on quality and safety, such as routine statistics and incident-reporting systems, are susceptible to such limitations (see, e.g., Currie et al., 2008; Waring, 2009). Accordingly, there is often a disjuncture between what is known informally and what reaches the status of official knowledge (Waring and Bishop, 2010). But this is not just a matter of a failure of communication: for some, there is a fundamental ontological difference between local knowledge (narrative, explanatory, and particular) and managerial knowledge (quantitative, predictive, and generalizing) that means that the former is not readily amenable to conversion into the latter (Hill, 2004; Yanow, 2004).

The ability to anticipate disaster is also stymied by the frailties of human cognition, as mediated by institutional context. Theorists of ‘sensemaking’ have shown how individuals' conceptualizations of organizations are permeated by shared understandings developed through communication with others (Weick et al., 2005). These sensemaking frames enable co-ordinated action within organizations (Tsoukas and Vladimirou, 2001), but can also blind managers to alternative understandings, resulting in collective myopia that can have disastrous results (Weick and Sutcliffe, 2003). How these frames might be disrupted is therefore of critical interest to those seeking to detect hazards and anticipate and avert problems.

Accordingly, calls for greater use of ‘soft intelligence’ deriving from sources beyond conventional metrics and formal knowledge-sharing systems have been made by academic and policy commentators alike (Dixon-Woods et al., 2014; Exworthy, 2010; Goddard et al., 1999; Millar et al., 2013). As Don Berwick and colleagues put it in a report commissioned by the British government in the aftermath of Stafford, “leaders need first-hand knowledge of the reality of the system at the front line, and they need to learn directly from and remain connected with those for whom they are responsible” (National Advisory Group on the Safety of Patients in England, 2013, p.15).

Yet how these calls for soft intelligence might be answered, and what challenges might lie in the way, remain little studied. A first step is to gain some conceptual purchase on terms that are often used interchangeably or treated as self-evident. In this regard, a useful (if somewhat heuristic) distinction can be made between data, knowledge or knowing, and intelligence. We propose that data represent the raw material of knowing, but need to be identified, selected, processed, interpreted, and made the basis of action. This implies, in Dretske's (1981) terms, a ‘knower’ capable of recognising the significance of soft data, of judging their importance and identifying those elements that may be especially valuable in revealing risks, and of using them to guide action. The knower (which may be an organizational entity rather than an individual) is likely to require social capacities (including an acceptance of the need to seek out discomfiting data) as well as cognitive capacities (including the ability to interpret, synthesise and judge). The knowledge generated must then be assessed for its ability to guide action. ‘Soft intelligence’, for our purposes, refers not to any discrete or prior entity, but to the *processes and behaviours* associated with seeking and identifying soft data on the part of this individual or organizational actor, and with the knowledge-producing activities of collation, synthesis, interpretation and application of insights.

In this article, we draw on evidence from a large interview-based study of senior stakeholders involved in quality and safety in the English National Health Service (NHS) with the aim of examining current and potential practice in relation to soft intelligence.

## 2 Methods

We conducted interviews with senior leaders and influencers as one element of a larger mixed-method research project on culture and behaviour in the English NHS (Dixon-Woods et al., 2014). Interview participants were purposively sampled from public sources and through extensive snowball sampling, and selected for their close involvement in quality and safety. They included NHS leaders in acute hospitals (e.g. chief executives, medical and nursing directors, executive and non-executive directors), senior frontline staff (e.g. clinical directors, lead nurses), non-clinical managers with roles in quality and safety (e.g. clinical governance officers), and individuals in commissioning, regulatory, policy, and academic organizations. In total, 107 participants were interviewed, including several with composite roles (Table 1). Interviews were transcribed verbatim. Ethical approval was granted by an NHS Research Ethics Committee (reference 10/H0406/38).

Our topic guide included questions about aspects of delivering quality and safety improvement in healthcare, including what participants understood the delivery of high-quality, safe care to involve, what was required to make it happen, and what approaches to diagnosing and addressing problems they used. We asked all participants about how they and others could *know* about the state of quality and safety in their organizations. From this, further discussion often flowed about the pros and cons of different approaches, the challenges of ascertaining knowledge of real problems, and the validity of different sources and how they could be reconciled. Building on these discussions, we adapted our topic guide to include a more explicit focus on use of sources of information beyond formal metrics and incident-reporting systems, which we operationalized by asking about “soft intelligence” and “soft data”—emic terms used spontaneously by some participants, which had apparent face validity for others. Our interviews took place between January and December 2011—a period when the public inquiry into Stafford hospital was never far from the news, and when the issue of patient safety in the NHS in general was a major focus of media interest.

GPM led the analysis for this paper, with an approach based on Charmaz's (2006) constructivist grounded theory. He read and reread transcripts, then identified all sections of interviews relating to how knowledge of quality and safety is produced, and how potential problems are identified. He undertook detailed coding of these sections, applying open codes inductively. These related in particular to the nature of the approaches described by participants, as well as their reported strengths and limitations. Next, he reread the coded excerpts sequentially (to view similarly coded data together instead of in the context of the original interviews), and considered them in relation to the wider literature on knowledge in organizations cited above. This process and discussion with colleagues led him to the sensemaking literature, and in light of this he embarked on a round of axial coding that resulted (*inter alia*) in the identification of three predominant approaches to soft intelligence, detailed below. Further comparison across transcripts and codes, alongside discussion among co-authors (who also had an intimate knowledge of the transcripts) facilitated identification of points of similarity and divergence between approaches, refinement of categories, and theorization about the strengths and weaknesses of different approaches. To the extent that it involved iterative cycling between data and the existing literature, our approach might be characterized as ‘abductive’ (Richardson and Kramer, 2006): our developing understanding of the data was informed by wider conceptual frameworks, particularly from organizational sociology and psychology. However, we also retained a strongly inductive logic throughout: for example, we constructed our descriptions of the three predominant approaches to soft intelligence through coding, comparing and recoding the interview data. Thus we used theory, as Timmermans and Tavory (2012, p.177) put it, “as a way to set up empirical puzzles” and view the data through alternative lenses.

The large number of interviews, and the consistency of emergent themes, gave us confidence in the relevance and validity of our findings. While our analysis was undertaken after data collection rather than alongside it—and so we cannot claim theoretical saturation in Charmaz's (2006) sense—our rigorous approach to coding, informed but not determined by the literature, and worked on by several authors, helped to ensure robustness.

## 3 Findings

Our findings are organized around three themes. First, we describe participants' views on the potential role of soft data, and their place in a system in which formal, quantitative metrics dominate. Next, we discuss the challenges of making sense of soft data. We describe three predominant approaches—which we label ‘Aggregation’, ‘Triangulation’, and ‘Instrumentalization’—used in making soft data comprehensible and actionable. Finally, we note some of the limitations of these approaches, and highlight some alternative approaches, which, though challenging, hold promise.

### 3.1 Soft intelligence: a crucial component of organizational vigilance

Across our sample, participants emphasised the difficulties for senior managers and leaders of forming an accurate picture of the quality of care delivered at the sharp end of care. Conventional approaches to trying to making the realities of the sharp end known to the blunt end of organizations (executives and board members) were sometimes slow and cumbersome, and prone to distort or diminish potential hazards.

*“What's actually happening day-to-day at ward level tends to be very invisible traditionally, remarkably so.”*

(Chief Executive)

Participants acknowledged these challenges explicitly, and offered accounts of how they sought to overcome them. They described multiple ways of collecting, collating and presenting ‘hard metrics’ using a range of different measures—such as daily statistics on process compliance, red-amber-green ratings, and safety ‘dashboards’—and sometimes bringing measures together in an attempt to gain more accurate and timely insights. At the same time, however, participants recognized that well intentioned efforts to expand metrics of quality could be counterproductive; in a system already dominated by a multitude of measures, extra ones could add to the noise without amplifying the signal:

*“It's almost a norm in the NHS: a problem comes along and our solution is to invent something to measure it and monitor it, and we don't say, ‘Right, what will we stop doing so we can do this?’ We say, ‘Here's another thing to do’, and we add it on, and then we add something else on, so there's no prioritizing or looking to see how can we amalgamate this with this.”*

(Associate Director of Nursing)

*“I love dashboards. I think they are great tools, but I am a bit concerned at the moment that the dashboards are getting so big, because we are trying to measure so many things that something is going to get lost in them.”*

(Deputy Director, Infection Prevention and Control)

Participants thus emphasised the need for supplementary data of a different kind. Though sometimes only upon specific prompting, many were agreed that soft data offered a critical counterpoint to the hard metrics yielded by audits, surveys and performance monitoring, and incident-reporting systems.

*“We do have areas that we have got shared understanding that we might have a concern about, so at the moment it would be like, we have had some problems in [operating] theatres, there is a problem on a certain ward. We are getting a sense: we have had a few different people mention problems in this area, so that's when we will [have] different people go and drop in with the shirt on and do the talking and try and get some softer data to see whether or not, what we are hearing or what the hard data is telling us, actually bears out in reality.”*

(Non-executive Director)

*“I think there's no substitute for those responsible for quality and safety to actually go around, talk to and make notes of those talks, with both patients and staff. Because that's the only way you find out about things.”*

(Medical Director)

Participants found that soft data offered rich, detailed, specific and highly pertinent insights into real or potential problems in quality of care, and in at least some organizations, these insights were taken seriously at senior levels:

*“People raise issues in all sorts of ways. I regularly get emails from all sorts of staff, saying, ‘I am concerned about this’, or, ‘Do you know about that?’”*

(Chief Executive)

*“I do it unofficially a lot, I bob about a lot and I have to go Ward B14 in a bit to pick something up and I try, if I'm there, to speak to the ward sister to pick something up, to speak to say three other people, whether it's the housekeeper, a nurse, and walk about. I say, ‘Good morning’, I say, ‘How are you doing today?’, I say ‘Are there any problems today, is there anything that's happened?’”*

(Patient Safety Manager)

Yet as we discuss next, while acknowledging the potential of such data, participants were also very conscious of their limitations—and this in turn had important consequences for how they did and did not use them.

### 3.2 Making data intelligible?

The richness of soft data was not in doubt. But if participants were conscious of the limitations of hard, quantitative data for monitoring quality and safety, then soft data were even more problematic. Participants' concerns related to the reliability, validity and evidential standing of the insights available from soft data. Issues raised spontaneously during ward visits might be matters of serious import or might equally be one-off ‘blips’, atypical of usual patterns of care. The issues raised might, participants suggested, owe more to the perceptions, motives or disposition of the individual raising them than to substantive risks to patient safety and care quality: a huge concern for one individual might turn out to be something trivial—or vice versa. Validation posed a major challenge: it was often very difficult for senior managers to distinguish the normal ‘moans and gripes’ of employees from serious concerns. With managerial time, energy and attention a scarce resource, identifying what needed action was crucial:

*“When I look at my soft intelligence where I might have one clinician that is telling me and moaning and groaning about an organization, it might be a one-off and one particular case and it could be that it is fact and widespread, so there is a judgment call.”*

(Chief Nurse)

To a degree, participants felt that staff could be relied upon to filter what they communicated based on their clinical knowledge and experience of expected standards of care. Patients and carers, on the other hand, were often seen to lack such a means of calibrating their concerns, and accordingly the pertinence and reliability of the insights they provided were seen as particularly variable. A recurrent concern was that patients and carers might be inclined to tolerate care that was in fact of poor quality.

*“[Patients] have got no benchmark to work to, so they have no idea of what quality the service should or shouldn't be, so they can't actually ascertain whether they're receiving a good or bad service.”*

(Improvement Consultant)

*“On the ward we were on for the elderly, two-thirds of them have got a degree of dementia: well they are not going to complain, are they? There is no way, so you really are reliant on their relatives and carers to raise concerns on their behalf, or staff. But I have to say, the staff, it is quite interesting because when you go on these visits, they will tell you how it is.”*

(Assistant Director of Quality)

In general, participants were clear that insights from the sharp end could not all be taken at face value: rather, active interpretive work was required to assess the validity, scope and importance of soft data, to make them intelligible, and to give them instrumental utility. Consistent with our construction of soft intelligence as a set of processes and behaviours, participants were clear that simply accessing soft data was not enough. They recognized the need to turn data into intelligence: to instil them with meaning, decouple them from the specifics of their production, and make them consequential as a tool for assessing the state of quality and safety.

Participants described these processes and behaviours in some detail, leading us to distinguish between three broad approaches to creating intelligence: ‘Aggregation’, ‘Triangulation’ and ‘Instrumentalization’.

1. *Aggregation.* When participants received a high volume of similar reports from multiple sources, they tended to conclude that the issue was more than a one-off incident or the idiosyncratic view of an individual complainant, that problems were real and persistent, and that they were worthy of investigation. Aggregation of this kind was therefore an important tool to distinguish data that merited action from those which could be safely sequestered:

*“I think that would have to be about patterns and trends: if you hear the same things coming up again and again.”*

(Patient Safety Manager)

*“I think patient stories are well underused personally. I think they are very powerful. [We] collate them and then the services will look at them and say, ‘Well we have got 10 patient stories and these are the key areas’, because you can't look at every single patient story and say that patient said that and that. So pulling out the key themes and trends and then saying, ‘What are our priorities from these?’”*

(Quality and Patient Safety Manager)

2. *Triangulation.* Second, participants often described using soft data in tandem with harder metrics, using one to validate the other. Soft data could prompt greater interrogation of quantitative data to identify temporal trends and cross-unit differences, or add nuance or richness to the scene drawn by quantitative data. Though participants placed greater confidence in the overall picture presented by the quantitative data, they reported that soft data could aid interpretation of the metrics, or suggest areas where better quantitative data collection was needed. But in this mode, the role of soft data was generally subordinate rather than having standing in its own right.

*“The way to distinguish is to do a bit of an attempt at triangulation, so if something is being suggested as being a problem, to actually look very hard to try and find the evidence that would support that. So if someone was complaining about poor levels of cleanliness, to actually look at infection rates for example or perhaps do a patient survey, do an audit and get patients' views [ … ] about what they feel about the cleanliness.”*

(Head of Patient Safety)

*“On the staff side of thing, as I said, the softer data really comes through again, the staff survey feedback twice a year, staff stories which we individually collect, and our visibility and assurances that we conduct with the board. [ … ] Again that's referred to in the board paper so you can go through that and the reports on that. So lots of soft intelligence to back up hard data”*

(Nonexecutive Director)

3. *Instrumentalization.* Finally, participants often described quite a different approach to utilizing data from patients and carers specifically, which, as noted above, they tended to see as poorly calibrated. Participants felt that the particular value of such data might lie less in diagnosis of problems relating to quality of care than in their ability to add emotional force to an argument premised on quantitative data, and thereby persuade others of the need for improvement. Soft data, in this mode, were mobilized instrumentally, as a ‘technology of persuasion’ (Armstrong et al., 2013).

*[Interviewer: Do you use soft measures as well as the hard data?] “Not as much as we should. So for example at the clinical effectiveness meeting where all our clinical directors meet once a month, our medical director told a striking patient story where harm resulted from the lack of a discharge letter, and it was effective and we should do much more of it than we do.”*

(Associate Director, Quality Improvement)

*“Engaging our senior leaders with a combination of data and patients' stories, walking them round, seeing the system issues around us. [ … ] I got our director of finance onto our wards and showed him the data, demonstrating numerically, and then stood at the end of some patients' beds and said, ‘Although I can show you the numbers that show we keep somebody in hospital this much longer than average compared to England, this is actually what it means to this patient today sitting in front of you’. And there were some magic moments there … when he turned to me and said, ‘These numbers come across my desk, but actually now I see what it means’.”*

(Clinical Lead for Quality Improvement)

Through these three translational mechanisms, participants described how they made soft data *meaningful*, and thus endowed them with function in relation to quality and safety, either diagnostically (Aggregation and Triangulation) or illustratively and persuasively (Instrumentalization). The three approaches to generating soft intelligence were not mutually exclusive, and two or more were often present in participants' accounts, with overlap evident between our categories of Aggregation and Triangulation. However, there were also important distinctions between the two: whereas Aggregation implied that soft data could become useful independently, through the accumulation of multiple soft data, Triangulation saw generating soft intelligence only as a complement to or means of validating data derived from conventional sources.

### 3.3 Beyond quantification and instrumentalization: valuing soft intelligence

Some participants also recognized, though, that in these processes of translation, something of the intrinsic value of softer forms of data might be lost—perhaps reproducing the inadequacies of formal metrics. If, for example, managers relied on Aggregation to isolate the most salient information offered by sources of soft intelligence, they risked disregarding important insights. This quantification of qualitative insights meant ascribing greater value to the views of the many rather than of the few, and risked neglecting the dissenting, but potentially valid, insights of those who demurred from the general view. Such views could, of course, be most important in exactly the situations where risks to quality and safety were greatest, particularly where substandard care had become normalized to the extent that staff were desensitized to deficits in quality and safety.

*“I went to visit my husband's aunt in hospital not long ago and the basic care she was getting was OK, but I was appalled by the approach of the nurses, the attitude of the nurses, absolutely horrified. And when I went to PALS [Patient Advice and Liaison Service—intermediary body providing advice and advocacy to patients], I said, ‘I am not making a complaint’, but there was so many alarm bells that I couldn't actually pinpoint and so I had to be general. PALS were quite defensive and I just thought: it is almost when you work in that environment, it becomes the norm, doesn't it, and whistle-blowing is not an issue because you don't even see a reason to whistle-blow.”*

(Clinical Governance and Quality Facilitator)

Such situations put the difficulties of interpretation associated with soft intelligence in a very different light. Rather than constituting an elusive, indeterminate resource whose diagnostic worth could only be gauged through quantification, it became a critical source of insight that other ways of knowing could not reveal, and which could not be reduced to metrics.

In this light, the idiosyncratic, uncalibrated views of patients or their carers became instead a fresh and untainted source of insight, not simply—as in Instrumentalization—a way of adding colour and human interest to dry numbers. Similarly, the implication of Triangulation—that soft data could be expected to ‘line up’ with harder metrics, providing a useful but epistemically subordinate complement to statistics—was also limiting. Taken together, exclusive reliance on these modes of translating data into soft intelligence risked painting a falsely reassuring picture. While Aggregation, Triangulation and Instrumentalization were useful means of deriving meaning from soft data, there was some recognition that reliance on such approaches alone risked overlooking their greatest potential value.

How, then, to apprehend and make use of this value? How to process such a detailed, frustrating, rich, and irreducibly complex resource? For some participants, the answer lay in improving the early parts of the process of generating soft intelligence, prior to interpretation—in what we label a greater ‘Systematization’ of processes. Rather than relying on concerns raised by staff, patients and carers themselves, they sought to generate mechanisms for soliciting these insights proactively, to eliminate the inconsistency associated with reliance on the decisions of self-selecting individuals to speak up on self-identified issues. Approaches included censuses of all patients at the point of discharge, and random sampling of staff or patients to obtain insights on topics of known importance to quality and safety:

*“One of the top hotels, [ … ] they invite on a random basis, people back to talk to the top team. We've mirrored that, by randomly selecting people who are in outpatient community services to meet with two execs to tell us what it was like, both good and bad. [ … ] Of all the random people we've invited, they've all come.”*

(Chief Nurse)

*“We have exit cards, so as they are about to go, they can complete an exit card which asks them, ‘What was the best thing about your stay? What is the one thing we could do better?’ What we are hoping to introduce, but have not done yet, is a card that could be used throughout their stay that just says, ‘What is the best thing that happened to you today?’, and, ‘What is the one thing that would have made your experience today better?”*

(Head of Nursing)

Such approaches sought to address some of the problems associated with *gathering* soft data. Random selection and census-based approaches could avoid the bias inherent in self-selection; solicitation could give voice to patients and carers who might otherwise remain silent; and up-front identification of topics of interest could give focus to soft data, while avoiding the generation of perceived irrelevancies. Systematizing approaches like this could ‘tame’ soft data at the point of collection, making them less idiographic and minimizing the risk of bias so that data were more readily useful:

*“I think there is a great resistance here to look at those, to look at gossip. It has to be returned, how shall we say? The views have to be in a controlled manner, within a controlled system.”*

(Chair of Board)

However, there was also a sense that this process too could transform the character of soft data. If part of their value lay in the way it could expose the unknown and unanticipated, then structuring soft data at the point of collection through Systematization to render them more tractable risked losing that value altogether. ‘Tame’ soft data offered a helpful way of obtaining a comprehensive and systematic picture of quality of care, but a better way of apprehending soft data ‘in the wild’—in all their context-laden, particular richness—was also needed.

Our interviews offered some intriguing indications of how this might be achieved. These involved finding novel ways of facilitating the generation and transmission of soft intelligence by encouraging staff, patients and carers to speak out about quality and safety, without structuring how they did it. These approaches, varied as they were, all hinted at the possibility of valuing soft intelligence for its untamed richness. They involved interpretation through dialogue, so that soft data were treated not as a static resource to be aggregated, triangulated or instrumentalized, but something more dynamic, whose value was better found in a joint process of meaning-making.

*“Some of our services have patient forums, up in our elderly care unit they have [ … ] listening clinics where the matrons actually invite patients or their relatives to come in and talk to them about their experiences and any issues, and the services, and what went well and what didn't go well, and that's a really good initiative up there.”*

(Lead Nurse, Infection Prevention)

*“We had an open day recently, which I though was really quite positive. And service users came in for a Saturday. [ … ] Our chief exec went out to a group of patient representatives and got feedback from the patients that had been in and out and said, ‘Look, what would you change? What went wrong? What do you want us to do?’ And that's been acted on.”*

(Nurse Specialist)

*“Our own local services will run user and carer fora. We have staff fora where they can give their views, and we seek those views. [ … ] We have instituted these conversations with our user groups and some of them have them quite regularly sort of weekly, formal meetings with people on the ward, et cetera, to say, you know, ‘What do you expect of us?’ And they will tell us in no uncertain terms, that the food isn't right, or the environment isn't right, people are rude—or they are very good, conversely!”*

(Non-executive Director)

Such approaches were labour-intensive and time-consuming. The insights they produced were not predictable or replicable. But in them, there seemed promise of a way of generating soft intelligence without taming its unpredictable richness.

## 4 Discussion

Our analysis suggests widespread agreement about the importance of soft intelligence among senior leaders of health systems in England, but much less consensus about how best to harvest value from soft data deriving from staff, patients, and carers. Participants in our study were not naïve about the limitations of relying solely on formal metrics to characterize the state of quality and safety at the sharp end of care. Rather, their struggles lay in how to access softer data and turn them into a useful form of knowing. Some of the most prominent strategies they used—those we label Aggregation (looking for accumulations of evidence) and Triangulation (synthesising various forms of evidence)—constituted interpretive filters that made it possible to identify potentially significant patterns. These approaches undoubtedly had utility, but they also risked reproducing some of the disadvantages of formal metrics: they meant that repeated and widely held views were given much greater credence than the exceptional views expressed by the few. *Reliability* of insights across multiple individuals was thus equated with *validity*, potentially neglecting rarely articulated but important insights. In consequence, serious problems with the quality of care might be missed: the broad-brush, aggregate picture produced by summary statistics might be reproduced, not corrected, by softer intelligence. An unchecked drift into failure (Dekker, 2012) might therefore occur not necessarily through failure to seek out soft data (though that may happen too), but rather because of defects in the processes and behaviours involved in generating soft intelligence.

The predominant strategies for making soft data intelligible that we found recall the cognitive limitations identified in the sensemaking literature as potential barriers to pre-emptive identification of imminent disaster (Weick and Sutcliffe, 2003; Weick et al., 2005). Sensemaking serves an important function in organization: it is a means of reducing the uncertainty, ambiguity, and cognitive dissonance caused by an abundance of conflicting data and a multiplicity of possible explanations (Alvesson and Spicer, 2012). But sensemaking is “not about truth and getting it right. Instead, it is about continued redrafting of an emerging story so that it becomes more comprehensive, incorporates more of the observed data, and is more resilient in the face of criticism” (Weick et al., 2005, p.415). Thus, while it may be highly functional in facilitating organizational processes in times of (apparent) normality, it can hinder free thinking and challenge. Reliance on Aggregation and Triangulation risks reinforcing a bias towards congruity and consensus, and resistance towards challenge and disruption. By seeking to strip soft data of their contextual specificity and translate them into more broadly applicable knowledge, these approaches give precedence to commonsense views that are plausible and broadly acceptable, over the difficult, counterintuitive, foreign—but potentially very useful—insights presented by a few iconoclasts. Systematization of data collection, similarly, might tend to emphasise the views of a majority whose organizational acculturation may also result in partiality. As such, these dominant approaches to soft intelligence risk reinforcing rather than confronting the potentially flawed understandings reached through reliance on conventional metrics and formal knowledge-management systems.

We do not argue that there is no place for such approaches: this kind of knowledge undoubtedly offers an important barometer of the overall state of an organization, and converting data into this kind of knowledge is a legitimate endeavour. However, soft data can also produce a different kind of knowledge, and relying solely on Aggregation, Triangulation and Systematization will not reap what is perhaps the most valuable potential element of the generation of soft intelligence: the alternative perspectives that provide a way to “challenge easy explanations” (Weick and Sutcliffe, 2003, p.82) offered by shared sensemaking frames—and thereby avoid the kind of narrowing of vision that may ultimately be implicated in disaster. Patients' and carers' views—prone to being relegated to illustrative use through Instrumentalization rather than recognized for their diagnostic utility—are arguably of particular value in this regard, since patients are not inculcated into the same sensemaking community. The same might be said of more junior clinicians, particularly those rotating between organizations (Keogh, 2013).

Generating this kind of soft intelligence, however, and avoiding reduction to common sensemaking frames, is far from easy. The unpredictable, untameable, spontaneous quality of soft data is what gave them their value, but it could be lost in efforts to systematize their collection and make them more tractable. Thus just as formal knowledge-management systems can corrupt or distort what is shared and becomes ‘official knowledge’ (Waring and Bishop, 2010), so too can efforts to make use of soft intelligence. For some, the very act of generating soft intelligence—collecting, interpreting and making use of soft data—is self-defeating. Hill (2004, p.227) describes the conversion of soft data into managerially useful knowledge as a process of “bring[ing] local knowledge in line,” so that it is “appropriated, redefined, and reformulated, [ … ] imbuing it with dominant meaning,” thus, in our terms, thwarting the processes and behaviours needed to generate soft intelligence that produces the greatest yield in improving safety and quality. Hill suggests that any effort to use soft data towards managerial objectives is doomed to failure, since it will inevitably obscure the richness of specific meaning with dominant sensemaking frames. Put this way, it is not the mediation of knowledge through formal knowledge-management systems that corrupts (cf. Currie et al., 2008), but the very desire to make managerial use of soft data.

We are not so pessimistic about the potential for soft intelligence. However, escaping Hill's negative prognosis requires a different approach to soft intelligence from those of Aggregation, Triangulation, Instrumentalization and Systematization. In plotting this escape, Schultze and Stabell's (2004) critique of knowledge management is helpful. Their analysis highlights the limitations of conventional approaches to knowledge management in organizations, which tend to characterize knowledge as an objective entity that can be separated unproblematically from the knower. Consequently, such approaches conceptualize knowledge management as an unproblematic process of accumulating more and more ‘pieces’ of knowledge with a view to “ultimate perfection and omniscience” (Schultze and Stabell, 2004, p.556)—in the manner of Aggregation—or reconciling knowledge from different perspectives within “systems of distributed cognition” (Schultze and Stabell, 2004, p.557) so that a progressively richer and more complete picture can be built—in the manner of Triangulation. Both these approaches assume that a realistic picture of an organizational feature—such as quality and safety—can be achieved, either through the cumulative piecing together of fragments of knowledge, or by reconciling different perspectives. However, deploying a dialogical understanding of knowledge, Schultze and Stabell (2004) demonstrate the flaws in such approaches. Consistent with Weick et al.'s (2005) notion of sensemaking, a dialogical perspective contends that knowledge is partial and inextricably tied up with dominant ideas about what is and what should be. In this light, argue Schultze and Stabell (2004, p.560), the particular benefit to be gained from efforts to gather and manage knowledge is not clarity, but disruption: “to create a space for multiple knowledges and marginalized voices [ … ] and to deconstruct self-evident concepts.”

Schultze and Stabell do not argue for a rejection of the benefits that can be derived from conventional approaches to knowledge management, as reflected in our constructs of Aggregation and Triangulation, but they do suggest the particular benefits to be derived from a dialogical approach. In our study we found glimpses of what such an approach might look like in healthcare, in the form of efforts to gather intelligence ‘in the wild’, untamed by Systematization, such as the forums for unstructured encounters between managers, patients and sharp-end staff described in the final three interview excerpts above. Others, too, have put forward helpfully practical suggestions about knowledge elicitation that offer clues about how a dialogical approach might be realized. Gavrilova and Andreeva (2012, pp.529–530), for example, discuss the particular merits of more ‘passive’ approaches to accessing intelligence that can give rise to “serendipitous revelation of pieces of tacit knowledge,” instead of reproducing the “already verbalized, formalized knowledge” that Systematization is likely to access. Again, this does not mean that there is no place for Systematization, but it does mean a place for the spontaneous soft data that can arise from chance encounters and unprompted discussions. Ideas like these hint at how managerial utility might be derived from soft intelligence in a way that values its multi-vocal richness, rather than contorting it to fit into conventional managerial frames. They also point to how soft intelligence may usefully be oriented towards more positive aspects of organizational functioning. Though a focus on latent troubles is valuable, relentless attention to the negative may not be productive.

## 5 Conclusion

The implications of our analysis are not easy. Deriving optimal benefit from soft intelligence requires more than simply accessing soft data; indeed, the ‘knowing’ produced through accessing such data may even be misleading, particularly if soft data are treated through the same interpretive frames as hard data. What matters is valuing soft data not only for their differences of scope and detail, but also when they offer dissent rather than confirmation. In particular, this means that while there is undoubtedly a place for the approaches to soft intelligence described most frequently by our participants, this needs to be leavened by an awareness that the overall pictures produced by these approaches can hide as much as they reveal. Where soft intelligence challenges the dominant picture, this should be valued as an opportunity rather than dismissed as an anomaly. As Macrae (2014, p.442) has it, “any fleeting uncertainties or doubts regarding patient safety—or current understandings of it—need to be seized upon and ruthlessly explored.” This aligns with the sense of ‘chronic unease’ said to characterize high-reliability organizations, where vigilance, propensity to worry, imagination, flexible thinking and even pessimism are seen as crucial components of an ability to anticipate problems (Fruhen et al., 2015). Used intelligently and sensitively, soft intelligence will be discomfiting and disruptive; often it will introduce greater doubt rather than greater certainty. But taking the challenge presented by soft intelligence seriously may equip healthcare managers with some of the tools they need to overcome predisposition towards the security provided by the superficial plausibility of dominant sensemaking frames—and perhaps thereby help them to evade the false reassurance that, as past experiences show, can breed complacency, ignorance, and undetected calamity.

## Figures and Tables

**Table 1:** Profile of participants.

Role	Number	%
Clinical manager	47	44
Manager	25	23
Clinician	15	14
Commentator (academic, thinktank, consultancy etc.)	13	12
Manager-commentator	4	4
Clinician-commentator	3	3
TOTAL	107	100
